# Bridging the sustainability gap in rural health equity: policy evaluation and transnational lessons from Guizhou’s targeted medical assistance program

**DOI:** 10.3389/fpubh.2025.1621223

**Published:** 2025-06-25

**Authors:** Kedi Sun

**Affiliations:** School of Graduate Studies, Lingnan University, Tuen Mun, Hong Kong SAR, China

**Keywords:** health poverty, medical assistance, lack of healthcare experts, Chinese medical assistance in Africa, Australian modified Monash model (MMM), stakeholder analysis

## Abstract

This study evaluated the sustainability of precision medical assistance policy in Guizhou Province through macro, meso and micro stakeholder analysis, combined with policy documents, statistical data and international comparison cases. The core objective is to identify barriers to long-term policy effectiveness and make evidence-based recommendations for improvement by drawing on global practice. This study used a three-tier stakeholder framework to analyze stakeholder dynamics at the macro level (national/local government), meso level (hospitals and medical teams in eastern Guizhou), and micro level (rural communities). Key findings reveal systemic sustainability challenges: at the macro level, unequal resource allocation and a “culture of dependency” on external assistance undermine local capacity building; At the meso-level, short-term personnel rotation (1–3 months on average), weak institutional management, and cultural mismatch (such as between the eastern team and Guizhou ethnic minorities) undermine service continuity; At the micro level, low policy awareness (due to poor communication in rural areas) and nonadherence to health behaviors (such as refusal to accept Western diagnoses in contrast to traditional practices) reduced the effectiveness of the intervention. Drawing on six decades of Chinese healthcare collaboration in Africa (with an emphasis on long-term capacity building and cultural integration) and Australia’s modified Monash Model (MMM) of rural talent retention (through hierarchical financial incentives and career pathways), the study proposes a three-dimensional framework: (1) local talent development (e.g., scholarship and rural career trajectories modeled on MMM); (2) Cross-regional collaboration for acculturation training (inspired by the pre-deployment immersion training for African medical teams); (3) culturally sensitive interventions (e.g., integration of traditional healers into primary care). These recommendations aim to provide actionable insights into health policy in resource-poor rural areas in China and globally, moving from short-term aid to strengthening sustainable local health systems.

## 1 Introduction

Against the backdrop of global health equity, the United Nations Sustainable Development Goals (SDGs), especially Goal 3 and Goal 10, have explicitly put forward the requirements for reducing poverty and promoting health equity on a global scale. Goal 3 aims to ensure a healthy lifestyle and promote the well-being of people of all ages, while Goal 10 is dedicated to reducing inequality within and between countries (1). The realization of these goals not only requires the joint efforts of the international community, but also demands that each country formulate corresponding policies and measures based on its own specific circumstances to address health inequality and poverty issues (1). Poverty is a significant problem in the world, and the United Nations has made it a priority in the Sustainable Development Goals (SDGs) (2). Guizhou had one of the largest poverty populations in China; a quarter of the whole population of Guizhou lived in poverty (3). In addition, Guizhou also had the largest number of population lifting out of poverty, with 9.23 million by 2020 (4, 5). Health poverty means poverty or returning to poverty due to illness; it occupies a third of the whole poverty population in Guizhou; therefore, the implementation of health poverty alleviation is of special significance for Guizhou against poverty (6, 7).

Health poverty is poverty or returning to poverty due to illness; it is an apparent problem. The shortage of medical personnel is one of the core problems of health poverty, which can be analyzed through multiple theoretical frameworks. First, from the application of stakeholder theory in health policy, as the core stakeholder, the shortage of medical personnel directly affects the supply and quality of medical services. This theoretical framework emphasizes that to solve this problem, organizational and policy changes should be promoted, and the synergy of multiple stakeholders such as the government, medical institutions, educational institutions and community organizations should be gathered (8). Equally important, the SDGs provide a framework for global health governance, particularly in promoting health and well-being. The SDGs advocate cross-sectoral collaboration and systems thinking to address social and health inequities. As the effectiveness of the health system directly depends on the reserve of medical personnel, the shortage of medical personnel has become an important bottleneck to achieve the Sustainable Development Goals. Therefore, a paradigm shift in institutional design is needed to more accurately identify, cultivate, allocate, and retain medical professionals (9, 10). Furthermore, social conditions as the root cause of health inequalities theory states that socioeconomic status (SES) forms a lasting association with health outcomes through resource endowments such as money, knowledge, and prestige. From this perspective, the shortage of medical talents is not only a human resource problem, but also a reflection of the imbalance of social resources allocation. The negative impact of medical talent shortage on health poverty can be mitigated by reducing inequalities in socioeconomic resources or developing more egalitarian interventions (11). Therefore, as the core problem of health poverty, the shortage of medical talents needs to integrate multi-dimensional frameworks such as stakeholder theory, sustainable development goals and social conditions theory, and achieve systematic governance through policy design and practical intervention from cross-theoretical perspectives.

To solve the problem, it is needed to explore the root problem using the 5 why model (12). Firstly, why does health poverty exist? The direct reason is that the medical costs are too high for them. Secondly, why is the expenditure so high for them? Because the poor population needs to afford high out-of-pocket expenditures (OOPE) on medical services (13, 14). Thirdly, why do they afford high OOPE? This is because the poor population needs to go outside the rural area to seek quality medical services. They would get a higher deductible but a lower compensation ratio if they had access to medical services in higher-class hospitals. The deductibles for primary hospitals, secondary hospitals, and tertiary hospitals are 100 RMB, 400 RMB, and 800 RMB, respectively, and the compensation ratios are 80, 75, and 60%, respectively (15). If they are hospitalized outside the Guizhou province to seek better-quality health care service, the deductible for different levels of hospitals would be 400 RMB, 800 RMB, and 1,500 RMB; moreover, the compensation ratio would be 75, 70, and 60% (15). As a result, the better health care service they are willing to receive, the higher OOPE they are required to afford, together with the other costs such as traffic, accommodation, and food when they stay away from their hometown for better health care services, further increasing their expenditure compared with the access to health care in their hometown. Fourthly, why do they need to go outside the rural area? This is due to the absence of quality healthcare services in their areas. Lastly, why is there an absence of quality healthcare services? The core reason behind this is the paucity of healthcare experts in their local medical facilities (16, 17). Consequently, the lack of healthcare experts would be considered the root problem of health poverty.

To begin, various pieces of literature have emphasized the importance of health poverty alleviation. Janjua and Kamal (18) and Atun et al. (19) emphasized the importance of improving the health status of the poor population for poverty alleviation. Dai et al. (20) highlighted that the government should focus on the poor population with poor health conditions. The papers from Lu et al. (21), Chen & Pan (22), and Wei et al. (23) collectively showed that health poverty alleviation could reduce the economic vulnerability of poor households, which means that it could largely prevent the possibility of catastrophic health expenditures. In addition, many papers have studied health poverty from different perspectives. Habtom et al. (24) studied the effect of medical financing on health poverty alleviation, taking Taijiang County in Guizhou Province as an example. They found that the China experience is good practice for other developing countries. Qin et al. (25), Liao et al. (26), and Zeng et al. (27) researched the impact of medical insurance on health poverty alleviation and suggested that the increase in compensation ratio could strengthen the medical condition of the poor population to reduce health poverty. Moreover, there are few papers examining the problems or challenges of health poverty alleviation. Guo (28) examined that the implementation of health poverty alleviation is insufficient and the policy lacks accuracy. Zhang et al. (29) argued that the health poverty alleviation project produced a positive effect; however, the fairness of the health poverty alleviation project. In summary, most literature related to health poverty has focused on the importance or achievement of the health poverty alleviation policy package as a whole. Although there are papers studying specific fields of health poverty alleviation, such as health insurance or health financing, and few studies have reported potential problems or challenges of health poverty alleviation, such as lack of fairness and lack of accuracy, a shortage of health care specialists is the root cause of health poverty, but misaligned incentives among policy makers, health care institutions and rural communities contribute to the persistence of the problem, and significant gaps in understanding remain. Specifically, little existing research has analyzed the dynamic interactions between macro-level policy design (e.g., resource allocation across provinces), meso-level implementation (e.g., short-term rotation of Eastern teams), and micro-level needs (e.g., low health literacy) that collectively affect policy sustainability. Therefore, through the stakeholder analysis of the precision medical assistance project in Guizhou Province, this paper intends to focus on the following questions: how to optimize the stakeholder coordination mechanism, solve the problem of health manpower shortage, and improve the policy life span? The following sections will assess the short-term outcomes of the policy, dissect the challenges to its sustainability from macro, meso, and micro perspectives, and draw lessons from international cases and propose feasible recommendations.

The second part is a literature review, combing the theoretical framework of health poverty and health manpower shortage, evaluating the effectiveness of China’s health poverty alleviation policy, and analyzing the sustainable medical assistance model in the world. The third section analyzes the policy environment of Guizhou’s medical relief program and its inclusion in China’s poverty alleviation strategy and the Healthy China 2030 Agenda. Section IV assesses short-term outcomes of the policy, including improvements in access to care and hospital capacity building. The fifth part analyzes the barriers faced at the national/local government level (macro), the hospital collaboration level in eastern Guizhou (meso), and the rural community level (micro) through the macro-meso—micro stakeholder framework. The sixth part draws on international cases—China’s long-term medical assistance to Africa and Australia’s modified Monash model—to put forward targeted policy recommendations. Finally, the last part integrates these findings, highlighting their implications for equitable rural health policy and global health equity research.

## 2 Literature review

### 2.1 Theoretical foundations: health poverty and healthcare workforce shortages

Health poverty and shortage of medical personnel are one of the major challenges in the field of health care. In order to better understand this problem, we can discuss it from the perspectives of “talent siphoning effect” and “urban and rural medical resource allocation theory.” Firstly, the “talent siphoning effect” refers to the loss of medical talents from rural areas to cities due to the better working conditions and living environment provided by urban areas. This phenomenon is common in rural areas of many countries, especially in economically underdeveloped areas. According to a study on the crisis of medical human resources in rural areas, there is a serious shortage of doctors in rural areas, mainly due to factors such as non-rural background, inadequate training, and imperfect incentive structure (30). In response to this problem, studies suggest that measures such as establishing rural medical schools, providing financial incentives, and changing curricula should be used to attract and retain medical talent. Secondly, the theory of urban and rural medical resources allocation emphasizes the unequal distribution of medical resources between urban and rural areas. Studies have shown that there are significant inequalities in the distribution of medical resources in rural areas of China, especially in the economically backward northwest areas. Despite improvements in recent years through medical reform and poverty alleviation policies, there is still a gap in the distribution of medical resources between poverty-stricken and non-poverty-stricken counties (31). In addition, the study also pointed out that although the equity of medical resources has improved, resources are still concentrated in more economically developed areas, which further aggravates the inequality of medical resources between urban and rural areas (32). Therefore, to solve the problem of health poverty and shortage of medical personnel requires multifaceted efforts, including policy support, resource allocation optimization, personnel training and incentive mechanism improvement. The comprehensive use of these strategies can alleviate the crisis of medical human resources in rural areas to a certain extent, and improve the accessibility and equity of medical services.

### 2.2 The effectiveness and limitations of China’s health poverty alleviation policy

China’s health poverty alleviation policy has achieved remarkable results in improving the quality of life and development conditions of the rural poor. However, existing studies also point to the limitations of these policies, particularly with regard to long-term sustainability. First of all, health poverty alleviation policy can effectively reduce the economic burden of the poor in the short term. For example, studies have shown that health poverty alleviation programs significantly reduce the vulnerability of rural households to poverty by reducing medical expenditure and improving the stock of human capital (23). In addition, health poverty alleviation policy has also played an important role in improving the health status and health equity of rural poor residents (20). These policies have helped many families escape poverty caused by illness by providing medical expense reimbursement and basic medical insurance, among other measures (33). However, although the health poverty alleviation policy has achieved some success in the short term, its long-term sustainability still faces challenges. Existing policies often focus on short-term assistance, while ignoring the cultivation of local talents and the building of long-term development capacity. This short-sighted policy design may lead to poor areas struggling to sustain their development gains after external aid ceases. Therefore, it is suggested that when formulating health poverty alleviation policies, more attention should be paid to the cultivation of local talents and the improvement of long-term development capacity to ensure the sustainability and effectiveness of the policy (34).

### 2.3 The effectiveness and limitations of China’s health poverty alleviation policy

China’s medical aid to Africa has always attracted the attention of the international community, especially with the promotion of the “Belt and Road” Initiative, China’s investment and aid in Africa have increased significantly. Such assistance is not only financial support, but also cooperation in the field of health care. China emphasizes “capacity-building orientation” in its medical assistance to Africa, which means that China not only provides material and financial support, but also focuses on improving the capacity of local medical personnel and the overall level of medical systems (35). In this context, Australia’s Modified Monash Model (MMM) provides an interesting reference mechanism. MMM is an incentive system for healthcare service delivery in remote and rural areas that aims to attract and retain healthcare professionals by providing financial and career development incentives. This “regional incentive mechanism” can provide enlightenment for Chinese medical aid in Africa, that is, how to enhance the sustainability and effectiveness of local medical systems through incentive mechanisms (36). By combining China’s “capacity building orientation” with Australia’s “regional incentive mechanism,” a more comprehensive aid strategy can be formed. This strategy focuses not only on short-term material and financial support, but also on long-term capacity building and system building to ensure the sustainability and effectiveness of aid. This combination strategy also helps to solve communication barriers and cultural differences that may occur in the aid process, and enhance the coordination and consistency of China-Africa medical cooperation (35).

Existing studies rely heavily on quantitative analysis of insurance data or policy evaluation at the macro level, ignoring the dynamics of stakeholder interactions at the micro level. In contrast, this study used a mixed approach, combining qualitative stakeholder interviews such as eastern medical team leaders, hospital managers in Guizhou Province with analysis of policy documents. No studies have empirically analyzed stakeholder conflict across levels (such as central government efficiency goals vs. Competition for resources by local governments) or the challenges of acculturation faced by eastern teams in multi-ethnic regions such as Guizhou. This study fills this gap by applying macro, meso, and micro stakeholder frameworks to uncover systemic barriers to policy sustainability.

## 3 Policy environment

The policy environment is to analyze under what context the policy was launched. To start with, based on the speech of President Xi, the Chinese government always put poverty alleviation as a priority in formulating policies and development plans, which emphasized that poverty alleviation should be put at the national level. In addition, the government would mobilize the entire country to support this matter (37). Moreover, from a broader aspect, common prosperity is the fundamental purpose of socialist development with Chinese characteristics; in such a context, poverty alleviation is an imperative way to achieve this goal (38). In addition, under the circumstances of the tight deadline for targeted poverty alleviation by 2020, the government needs to find an efficient way to accomplish the goal. Furthermore, public ownership is the mainstay of the socialist market economy (39). Therefore, the government was very powerful in mobilizing social resources, and as a result, public institutions could respond rapidly to the government. Lastly, President Xi has also put forward the Health China 2030 program, which put health at the center of the policy-making process in China and indicated that the government would increasingly invest in the healthcare industry. Overall, these factors collectively boosted the launch of the assistance policy (40).

## 4 Targeted medical assistance policy from eastern hospitals

To handle the lack of healthcare experts in the poor area of Guizhou, the Chinese government proposed the targeted medical assistance project in 2016 requiring the tertiary hospitals in eastern cities, including Shanghai, Dalian, Suzhou, Hangzhou, Ningbo, Qingdao, Guangzhou, and other 7 eastern cities, to provide long-term assistance for 7 hospitals in cities and 37 hospitals in poor counties in Guizhou, respectively (7). By the end of 2019, the number of assisting hospitals from eastern cities had increased to 266 at different levels. With their help, over 40 medical disciplines were constructed, such as severe medical disciplines, emergency medical clinics, and cardiovascular medicine (41). As a result, the rate of people needed to transit to the upper level of hospitals fell from 5.43% in 2016 to 2.47% in 2018 (41). Overall, this policy indeed had positive outcomes in the short term through direct medical expert assistance.

## 5 Discussion

### 5.1 Sustainability challenges from stakeholder perspectives

The objective of the targeted medical assistance policy was to establish a long-term sustainable relationship of targeted medical expert assistance between eastern advanced hospitals and underdeveloped hospitals in Guizhou (41). Although, based on the policy outcome stated above, this policy had positive outcomes in the short term, it is noticeable that the policy lacks sustainability in many cases. This part will examine the reasons why it lacks sustainability from stakeholder perspectives at the macro, meso, and micro levels.

#### 5.1.1 Macro level (national government, Guizhou government)

Firstly, when it comes to the macro level of the policy, the stakeholders involved are supposed to be the national government and the Guizhou government. The negative implication would be the inequity of the assistance because the policy cannot ensure all the poor areas get the same assistance; some areas may obtain more resources than other poor areas in Guizhou, which may lead to other social problems (42). Such aid may foster an overdependence on external support, similar to the “dependency culture” proposed by Schneider and Jacoby (43), who argue that continued welfare policies undermine the self-sufficiency initiative of recipients. In the Guizhou context, some counties prioritize short-term medical team deployment over investment in local medical schools, reflecting the passive dependence observed in the social welfare system. This dynamic undermines the long-term sustainability of the policy, as local institutions lack incentives to build autonomous medical capacity. Additionally, there may be discrepancies in the evaluation standards and methods of medical assistance in Guizhou among different assisting teams; the evaluation results may be inaccurate and incomplete; consequently, the actual outcome of the assistance may not be objectively reflected. Lastly, long-term eastern medical assistance may cause a paucity of local medical resources.

#### 5.1.2 Meso level (eastern teams, hospitals)

Secondly, referring to the meso level of this policy, eastern hospitals and Guizhou hospitals are recognized to be the main stakeholders. Due to the backward development of Guizhou’s medical resources, the available support, which may include supporting teams and medical equipment for the eastern experts, would also be restricted, which may impede the outcome of the policy (44). Moreover, given that the eastern teams come from different regions and hospitals, there is a certain difference in the level of medical skills or customs among them, which may affect the quality of medical treatment (45). In addition, the short duration of the eastern team in Guizhou and the frequent shift of personnel may led to the instability of the local medical team; for example, the minimum requirement of duration is only 1 month for Huizhou city of Guangdong medical team to assist Guizhou, which is not conducive to the improvement of the local medical techniques (46). Furthermore, the assisting medical team are not employed in the Guizhou hospitals, hence there is a lack of management and supervision of them. Another factor is the lack of motivation. To a certain extent, the intrinsic motivation of Eastern assistance is driven by the assigned tasks from the central government and the relation between the targeted medical assistance and the promotion of professional titles or other incentives. It was stated in the policy document launched by The Medical Administration Hospital Authority of China (47), indicating that the outstanding assisting health workers shall be given priority in the promotion of professional titles, position mobility, promotion and appointment, and various evaluations of honors and awards. As a result, their efficiency and performance may not be as high as expected. Lastly, Cultural differences further hinder the effectiveness of the policy. As documented by Gao (57) in a study of Yi communities in northwestern Guizhou, medical teams in the east often clashed with local customs, such as traditional herbal rituals. For example, some Yi patients refused Western-style diagnosis without understanding its purpose, while physicians misinterpreted cultural taboos regarding blood tests as non-compliance. With an average deployment time of only 1–3 months, teams lack time to address this misconception, resulting in mistrust and low utilization of medical services.

#### 5.1.3 Micro level (local poor population)

Thirdly, regarding the micro level, the local population should be the most relevant stakeholder. Despite the increasing medical assistance, the local population may not know it due to the poor network communication in the Guizhou rural area, which would decrease their accessibility to the assisting team. In addition, on account of poor health awareness, some healthcare projects like health promotion or health check activities cannot fully make a result as expected. For example, a study conducted by Zhang et al. (48) illustrated that the rural population did not cooperate with the local healthcare projects and was not satisfied with them, because they thought they needed to spend a long time and walk a long distance to access the healthcare services to do a useless health check without any subsidy. Therefore, for the medical assisting team, the local population is probably unable to take full advantage of these resources.

### 5.2 Lessons from international experiences

Overall, from a stakeholder perspective at the macro, meso and micro level, it can be notable that the medical assistance policy has various negative implications and obstacles which would lead to a non-sustainability of the policy, furthermore, other social problems may occur. Therefore, it is necessary to make policy amendments for the long-term success.

#### 5.2.1 Experience from Chinese medical assistance in Africa

There are many successful medical assistance projects around the world. One of them is the Chinese medical teams in Africa, which is a long-term cooperation between China and poor areas in Africa implemented in 1963 in Algeria (49). This program has made a great achievement in the long term, it has been continuously operating for 60 years, more than 20,000 medical experts have been dispatched to 51 African countries, and over 270 million local population has been served before 2017 (50). There are 4 main experiences of the program worthy of reference. Firstly, the professional team, each Chinese medical team is constituted of senior professionals, junior physicians, nurses, leaders, translators and chiefs (50). Therefore, the members of the team can do their jobs well to produce a good collaboration, which would increase the efficiency of the team as a whole. Secondly, active integration into the local culture, each team would be trained for 4–6 months before they commence the assistance, including the local culture, language, etc. (51). In addition, each medical team would live there for approximately 2 years for tenure and the National Health Commission allow and encourage them to go with their spouse for the longer term stay, which would help them fully learn and integrate into the local culture, as a result, the outcome of the assistance would be boosted (50, 51). Thirdly, strong intrinsic motivation, due to the humanitarian spirit and the sense of mission on behalf of the state, the Chinese medical team has always exhausted their ability to help the local poor population, for example, the health promotion to increase the health awareness of the local people, which would enhance the cooperation between them, and further won the high praise by the local government and residents; hence, their performance would be enhanced (52). Fourthly, systematic evaluation system, the program has a comprehensive evaluation system for monitoring the medical teams from 4 aspects that are Virtue, ability, diligence, and achievement, made by medical teams themselves, original hospitals, embassies and consulates, and health authorities, collectively (51). As a result, the progress of the policy would be visible and adjustable according to the drawbacks timely. For example, in the early stage of the 2000s, the focus of medical assistance in Africa was basic health workers without a diversity of subjects, after 2004, the focus was shifted to the construction of infrastructure, and after that from 2006 to 2009, wide and in-depth control for malaria was the main task, and the focus changed to infectious diseases like Ebola from 2011 to 2014, all the changes and shifts of medical assistance in Africa were based on the evaluation of the performance of the medical teams and status quo (53).

#### 5.2.2 Experience from Australia

About a quarter of the Australian population live in rural areas, where people also face challenges in accessing quality healthcare services, people living in remote areas are more likely to get diseases and have a lower average expected age in comparison with those living in the inner cities, due to the lack of healthcare experts (54). To increase the number of local healthcare experts, the Australian Government designed a human resource formulation system based on the Modified Monash Model (MMM) to facilitate talent cultivation and retention rather than medical assistance from other cities in 2015, it classified cities within Australia from MM1 to MM7 based on the remoteness of the city, specifically, the MM1 areas are big cities, while the MM7 areas are very remote communities (36, 55). On account of the MMM, the more remote the city is, the more incentives and supports would be given to the medical workers to motivate them to settle down and work in the very remote area, and the Australian government would review and adjust the classification every 5 years (55). This policy also worked well in quite the long term, the increase in the number of healthcare workers in the MM7 area was the highest compared with that in other areas (56). Three reasons lead to the sustainable success of the policy. Firstly, It provided more career support for remote healthcare workers, the Remote Vocational Training Scheme (RVTS) that provided structured online medical education and training were only available to the people of MM4-MM7, moreover, the special remuneration incentives of a total of 500,000 AUD in 3 years was only available to the people of MM5-MM7 (56). In addition, the physicians working in the MM3-MM7 can apply General Practitioner Procedural Training Support Program (GPPTSP) to get a certificate and scholarship of 40,000AUD, and people living in the MM3-MM7 can apply for the scholarship from the Health Workforce Scholarship Program (56). Secondly, it relaxed the entry requirements for remote healthcare workers, allowing non-registered healthcare workers to provide service after working hours (56). Thirdly, it encourages more international medical students to work in rural areas, for example, the length of required medical services for those in very remote area was 5 years, while those living in main cities was 10 years (56). Thus, it can be concluded that the healthcare workers living in the remote area can get more career support, therefore, they would be more willing to settle down and work in the remote areas. This policy emphasizes the cultivation and retention of local healthcare workers, which is why it can lead to a long-term and sustainable increase in the number of healthcare workers.

In general, the success of China’s six decades of medical cooperation in Africa has benefited from its emphasis on cross-cultural capacity building. Medical teams are trained in local language and customs for 4–6 months prior to deployment (51) and work with host countries to establish local clinics suitable for transnational and cross-cultural contexts. For example, in Algeria, Chinese doctors adapted to local preferences for community medical consultation, improving acceptance and sustainability. “In contrast, Australia’s Modified Monash model (MMM) addresses domestic Labor inequality through institutional incentives. The division of areas into MM1-MM7 based on their degree of remote, the policy provides targeted bonuses (up to AU $500,000 for MM7 employees) and simplified career pathways for rural specialists -strategies that work in a unified national healthcare system. Unlike the cross-cultural challenges in Africa, MMM used the existing administrative structure to retain local talent, making it more applicable to the domestic environment in Guizhou.

#### 5.2.3 Policy optimization pathways

Based on the case study from Africa and Australia, policy recommendations for medical assistance policy in Guizhou could be proposed at macro, meso, and micro accordingly. Firstly, at the macro level, the government should focus on talent cultivation rather than short-term assistance to keep a sustainable healthcare human resource. They should provide more educational programs and financial support for local healthcare education, and encourage the eastern team to settle down in the Guizhou hospitals where they may get better career development compared with working in the hospitals in the eastern cities. In addition, they should establish a comprehensive evaluation system for a timely adjustment of the priority of assistance. Secondly, at the meso level, the government should enlarge the assistance duration to reduce the friction cost of frequent work handovers. Moreover, the government should enhance the training for the assistance team to increase their cultural adaptability and motivation. Equally important, the government should improve the management of medical teams through the cooperation of the hospitals in Guizhou and eastern cities. Lastly, at the micro level, the Guizhou government should enhance the promotion of the eastern teams and the health knowledge to the local poor population to increase the accessibility of the poor population to healthcare services.

## 6 Conclusion

Through stakeholder analysis and international comparative studies, this study systematically evaluates the sustainability of medical assistance policies in Guizhou Province and identifies key barriers to their long-term effects. The existing literature on health poverty alleviation mainly focuses on policy outcomes or economic implications at the macro level, while ignoring the agency of health care workers in rural Settings and the role of acculturalization in cross-regional cooperation. Research reveals a dynamic interplay between macro-meso-micro barriers: policy design deficiencies at the macro level (e.g., resource inequity, dependency culture) can exacerbate implementation inefficiencies at the meso level (e.g., short-term staff rotation, cultural mismatch), which in turn can impair community engagement at the micro level (e.g., low policy awareness, noncompliance with health behaviors). To address these challenges, this study proposes a three-dimensional framework that integrates indigenous talent development (learning from Australia’s modified Monash Model), cross-regional cooperation and acculturation training (learning from China’s long-term medical assistance to Africa), and culturally sensitive intervention (such as integrating traditional healers). This framework not only provides an actionable guide for Guizhou, but also enrichs global health policy by highlighting the need to align stakeholder incentives with cultural context in low-resource Settings. Future research could continue to conduct longitudinal studies to quantify the impact of these reforms on talent retention or compare the acculturation strategies of different ethnic communities in Guizhou to refine specific contextual solutions.
